# Bridging the Gap: PSMA Radioligand Therapy for Asian Men with Prostate Cancer

**DOI:** 10.1007/s13139-025-00946-w

**Published:** 2025-08-26

**Authors:** Minseok Suh, Joo Hyun O

**Affiliations:** 1 Departments of Nuclear Medicine, Seoul National University Hospital, Seoul National University College of Medicine, Seoul, Republic of Korea; 2 Division of Nuclear Medicine, Department of Radiology, Seoul St. Mary’s Hospital, The Catholic University of Korea, Seoul, Republic of Korea

**Keywords:** PSMA, Prostate cancer, Asian, Radioligand therapy, Ethnic disparity

## Abstract

**Abstract:**

Prostate-specific membrane antigen (PSMA)-targeted radioligand therapy (RLT) is transforming the treatment landscape for prostate cancer. However, clinical data and outcomes for Asian patients remain limited and they are underrepresented in pivotal clinical trials. Although emerging retrospective series from Asia demonstrate comparable efficacy and safety profiles to Western cohorts, several points warrant attention. Asian men frequently present with aggressive, advanced-stage disease and distinct genomic profiles, characterized by lower rates of ERG fusions and TP53/PTEN mutations, and higher frequencies of FOXA1 and SPOP alterations. These genomic differences could influence PSMA expression and responsiveness to therapy. Additionally, Asian populations exhibit higher susceptibility to myelosuppression, necessitating careful patient selection and dose management. The development of region-specific radiopharmaceuticals and clinical trials, alongside systematic real-world data collection, will be essential for optimizing PSMA-targeted RLT in Asian contexts. Incorporating PSMA-based therapies earlier in disease management strategies also presents promising avenues for improving outcomes. Personalized approaches informed by ethnic-specific disease biology and clinical experience will be crucial for effectively bridging the current knowledge gaps in PSMA-targeted RLT for Asian prostate cancer patients.

**Clinical trial number:**

Not applicable

## Introduction

Prostate cancer (PCa) was the most common malignancy diagnosed in men in 2024 and 2nd leading cause of cancer death worldwide​ [1]. However, its incidence and clinical courses vary significantly across different ethnic and racial groups [2]. For example, men of Asian ancestry have historically had lower recorded PCa incidence and mortality than Western populations, though this gap has been narrowing in recent years [2–4]. In a migration study, Asians who relocated to the United States exhibited higher PCa incidence compared to those in their home countries, but their rates still remained significantly lower than those of non-Hispanic whites in the same environment [5]. Notably, Asian patients often present with more advanced or high-grade disease despite lower overall incidence [6]. Studies of men in the United States have also shown that Asian American patients (e.g., of Chinese or Japanese origin) are more likely to have unfavorable risk features at diagnosis compared to non-Hispanic whites​ [7]. Nonetheless, after adjusting for clinical factors and treatment history, Asian ancestry is associated with significantly better PCa-specific survival than white ancestry​ [7]. This seemingly contradictory survival advantage, despite worse pathology at diagnosis, suggests that disparities in PCa outcomes are not solely attributable to differences in healthcare access or screening practices [6]. Evidence indicates environmental factors and underlying biological differences play important roles in the ethnic disparities of PCa [8–10].

With the advent of prostate-specific membrane antigen (PSMA) targeted diagnostics and therapeutics, there is a growing need to understand how such ethnic differences could figure into the latest treatment scheme. PSMA-targeted radioligand therapy (RLT), exemplified by lutetium-177–labeled PSMA ligands, has emerged as a valuable option for metastatic castration-resistant PCa (mCRPC). The pivotal VISION trial demonstrated significant survival benefits with [^177^Lu]Lu-PSMA-617​, leading to regulatory approvals [11]. Given the known ethnic disparities in the biology of PCa, this underrepresentation of Asians raises questions about the generalization of trial results and whether Asian patients may experience different treatment outcomes or side effects. To date, clinical data on PSMA-targeted RLT in Asian populations are limited, coming mostly from small retrospective series. In this review, we summarize the known racial and ethnic differences in PCa biology with a focus on East Asian populations, and discuss current evidence and considerations for PSMA-targeted RLT in these patients.

### Clinical Efficacy and Safety of PSMA-targeted RLT in Asian Cohorts

PSMA-targeted RLT has rapidly become a promising treatment modality for advanced PCa. Multiple trials have validated its efficacy in predominantly Western populations. The international phase III VISION trial (> 800 patients) established the superiority of [^177^Lu]Lu-PSMA-617 plus standard of care over standard of care alone in men with heavily pre-treated mCRPC​. Specifically, PSMA-targeted RLT improved the median overall survival (OS) by about 4 months (15.3 vs. 11.3 months) and significantly prolonged the radiographic progression-free survival (rPFS) to 8.7 months, compared to 3.4 months with standard of care alone [11]. Another landmark study, the TheraP trial (ANZUP 1603) from Australia and New Zealand, was a head-to-head comparison of [^177^Lu]Lu-PSMA-617 versus cabazitaxel chemotherapy in mCRPC after docetaxel [12]. TheraP demonstrated markedly higher prostate-specific antigen (PSA) response rates with PSMA-targeted RLT – two-thirds of patients achieved ≥ 50% PSA decline with [^177^Lu]Lu-PSMA-617, compared to 37% with cabazitaxel. Importantly, [^177^Lu]Lu-PSMA-617 was also associated with a significantly better safety profile, with fewer grade 3–4 adverse events (33% vs. 53%) and improved patient-reported outcomes, including less fatigue and fewer treatment-related side effects. These results cemented PSMA-targeted RLT as an effective option after androgen receptor pathway inhibitors (ARPI) and chemotherapy. However, it must be noted that Asian patients were sparsely represented in these trials. Only 2.4% of the accrued VISION population consisted of Asian patients, and the majority of TheraP participants were White without further analysis by the racial groups. In the subgroup analysis of VISION, Asian race was the only subgroup which did not favor [^177^Lu]Lu-PSMA-617 + standard of care over standard of care alone for PFS (Asian *n* = 15) and also for OS (Asian *n* = 20). However, this finding must be interpreted with caution due to the very small sample size, which limits the statistical power to detect treatment effects in this subgroup.

Initial clinical outcome of PSMA-targeted RLT in Asian populations is beginning to emerge, largely from retrospective reports. A systematic literature review in 2022 identified only a handful of studies reporting on PSMA-targeted RLT outcomes specifically in Asian patients [13]. A recent single-institution study from Singapore analyzed 84 mCRPC patients treated with [^177^Lu]Lu-PSMA-I&T and found the therapy to be both effective and well-tolerated, with outcomes “mostly similar to their global counterparts”​ [14]. In that cohort, 52% of patients achieved a ≥ 50% PSA decline on therapy, and those responders had significantly prolonged OS and PSA PFS compared to non-responders​. The median OS was 12.2 months, which is slightly shorter than the 15.3 months in VISION, but patients in the Asian series had much higher disease burden at baseline​. Indeed, 77% of the Asian cohort had > 20 metastatic lesions and over half had disseminated bone metastases (superscan pattern)​, a scenario that precluded enrollment in the VISION trial. This heavy tumor burden may account for the numerically shorter OS, rather than any ethnic difference in drug efficacy. Reported toxicities in the Asian population mirrored those reported in Western studies, and myelosuppression was the dose-limiting side effect, and mild xerostomia was seen in a small fraction of the patients​. Prospective data from China further supports these findings. In the first reported [^177^Lu]Lu-PSMA-I&T trial in East Asians, 40 mCRPC patients (35 Chinese, 5 Korean) received up to 5 cycles (median ~ 2 cycles) at 3.7–14.8 GBq per cycle [15]. PSA responses were robust, with 62.5% of patients achieving a PSA decline consistent with partial response​. Hematologic toxicity was observed in 70% of patients, though predominantly grade 1–2; and all were resolved or manageable​. No unexpected safety signals emerged. Factors associated with poorer treatment outcomes in this mostly Chinese cohort included high baseline alkaline phosphatase, lactate dehydrogenase and aspartate aminotransferase levels, and being overweight​, suggesting that patients with very extensive disease or impaired liver function might have more guarded responses. In addition to East Asian experiences, PSMA-targeted RLT has also been explored in other Asian regions. In a single-center study from India involving 40 heavily pre-treated mCRPC patients, [^177^Lu]Lu-PSMA-617 therapy achieved symptomatic or biochemical responses in over half the patients, with an imaging response rate of 43% and a median OS of 12 months [16]. Treatment was generally well tolerated, with only low-grade hematologic toxicity and mild xerostomia reported. Complementing these findings, a larger Indian cohort study (n = 121) reported durable responses with PSA declines in 73% of patients and a median OS of 16 months after a median of three treatment cycles [17]. The most common adverse events were mild fatigue, nausea, and dry mouth, indicating favorable tolerability even with long-term follow-up. In Iranian cohorts, PSMA-targeted RLT was associated with notable PSA declines, pain relief, and preservation of functional status, with no unexpected toxicities observed [18, 19]. These studies suggest that PSMA-targeted RLT may provide consistent therapeutic value across diverse Asian populations. Reports so far support PSMA-targeted RLT to be effective and generally well-tolerated in Asian mCRPC patients, with outcomes broadly aligned with those reported in previous global trials. However, given that current evidence is largely based on small, retrospective cohorts, it remains uncertain whether subtle ethnic or biological differences could influence the treatment response or safety profile in a broader clinical practice.

### Consideration for PSMA-targeted RLT in Asian Cohorts

Despite comparable outcomes observed to date, several important factors should be considered when applying PSMA-targeted RLT in Asian patients, including differences in tumor aggressiveness, genomic profiles, and potential variations in treatment tolerance.

1. Aggressive Disease at Presentation
  Several studies suggest that Asian patients are more likely to present with aggressive features of PCa at diagnosis, including higher PSA levels, advanced stage, and higher Gleason scores compared to Western populations [2, 4]. Dong et al. reported that East Asian men had nearly double the proportion of Gleason ≥ 8 tumors on biopsy compared to Western men [6]. The authors suggested that this difference was primarily due to delayed diagnosis, as PSA-adjusted analyses attenuated the disparity. While this interpretation emphasizes screening practices, differences in underlying biological characteristics were supported by data from Asian-American cohorts. Even within the US healthcare system, Asian men are more frequently diagnosed at advanced stages compared to Caucasians, suggesting that ethnic factors could contribute to the manifestation of PCa at diagnosis [7]. Whatever the cause may be, aggressive or advanced disease at presentation is a feature of Asian PCa patients, and underscore the need for optimizing the diagnostic methods and early therapeutic interventions.
  In this context, PSMA PET/CT emerges as an essential tool for accurate staging and risk stratification in Asian PCa patients. To be fair, the diagnostic performance of PSMA PET/CT has not be extensively analyzed by racial groups, but the clinical experience in the past few years in Korea support the value of PSMA PET/CT for staging and recurrence detection. Given the higher likelihood of aggressive and advanced disease at diagnosis, conventional imaging modalities such as CT and bone scans can underestimate the true extent of metastatic spread. PSMA PET/CT offers superior sensitivity in detecting both typical and atypical metastatic lesions that would otherwise have gone unnoticed [20, 21]. This is critical for guiding treatment decisions in high-risk patients, where early identification of metastatic burden can influence the choice and timing of systemic therapies [22, 23]. Moreover, PSMA PET/CT can help differentiate patients who may benefit from intensified treatment approaches, including consideration of early PSMA-targeted RLT.
  Building upon its diagnostic value, one emerging approach is the earlier integration of PSMA-targeted RLT in the treatment pathway. PSMA-targeted RLT was initially reserved for patients with mCRPC after exhausting multiple lines of therapy, as demonstrated in the VISION trial. However, recognizing the potential benefits of addressing metastatic disease before resistance mechanisms develop, recent clinical trials have investigated its use in chemotherapy-naïve settings. In a randomized phase 2 trial, [^177^Lu]Lu-PSMA-617 demonstrated non-inferior PSA response rates (60%) compared to docetaxel (40%) in chemotherapy-naïve mCRPC patients, with better quality-of-life outcomes and fewer grade ≥ 3 adverse events [24]. The final survival analysis confirmed comparable median OS between the two arms (19.0 vs. 15.0 months, per-protocol analysis) [25]. Furthermore, the recent PSMAfore Phase 3 trial compared [^177^Lu]Lu-PSMA-617 to ARPI switch in taxane-naïve mCRPC patients and showed a significant improvement in rPFS (11.6 vs. 5.6 months; HR 0.49), with fewer grade 3–5 adverse events [26]. These studies support the feasibility and clinical benefit of using PSMA-targeted RLT earlier in the disease course. Building on these findings, the ongoing Phase III PSMAddition trial (NCT04720157) is evaluating whether adding [^177^Lu]Lu-PSMA-617 to standard androgen deprivation therapy (ADT) and ARPI in metastatic hormone-sensitive PCa (mHSPC) can improve patient outcomes [27]. Similarly, the PSMA-DC trial (NCT05939414) targets patients with oligometastatic PCa, aiming to delay disease progression and defer initiation of ADT through early use of PSMA-targeted RLT following stereotactic body radiation therapy. Both trials reflect a paradigm shift toward more proactive management strategies, where PSMA-targeted RLT is introduced to patients who remain naïve to chemotherapy or long-term hormonal therapy. For Asian patients—who often present with aggressive, high-burden disease—these early-intervention approaches could offer significant advantages by achieving better disease control before tumor clones refractory to treatment emerge. While these strategies remain investigational, they highlight how PSMA PET/CT can be incorporated to personalize the therapeutic approach in high-risk populations.
2. Genomic Disparity
  Asian PCa exhibits distinct genomic profiles compared to Western cohorts [4, 9, 10]. Notably, East Asian tumors have a lower prevalence of the common ERG gene fusion: 21–25% of East Asian patients show ERG positivity, compared to approximately 50% in Western or South Asian populations [4]. Consequently, Asian cases are often enriched in ERG-negative molecular subtypes, such as SPOP or FOXA1 mutations [4, 9]. A large-scale sequencing study of Chinese patients [10] reported FOXA1 mutations in 11.4% of localized tumors, whereas Western cohorts showed a prevalence of 3.8%. In contrast, TP53 mutations were identified in 5.1% of Chinese patients versus 12.8% in Western patients. Similarly, at the metastatic hormone-sensitive stage, Chinese patients exhibited fewer alterations in TP53 (11.3% vs. 29.4%), PTEN (2.5% vs. 10.3%), and APC (0.8% vs. 5.9%) compared to Western counterparts. In the IPATential150 trial, PTEN loss was observed in 15% of East Asian men with mCRPC, notably lower than the 31.3% from non-Asian regions, highlighting the regional disparity in PTEN alteration rates during disease progression [28]. These genomic differences may explain the existence of inherent biological variability by ethnicity.
  Altered androgen receptor (AR) signaling pathways, and distinctive patterns of tumor suppressor gene alterations likely drive the differences in pathophysiology, potentially affecting the expression of therapeutic targets such as PSMA.PSMA expression is known to be influenced by multiple factors, including the AR signaling activity and the tumor’s differentiation status [29, 30]. Preclinical studies suggest that active AR signaling may suppress FOLH1 (PSMA) expression, whereas androgen deprivation or AR blockade can lead to upregulation of PSMA levels [30]. Clinically, this has been observed in patients initiating ADT, where PSMA expression often increases, particularly in tumors with low baseline PSMA uptake [31–33], though these upregulations are not universally reproducible [34, 35]. In Asian PCa, where mutations in FOXA1 and SPOP are more common and losses of TP53 and PTEN occur less frequently, AR-driven regulation may remain relatively intact during early disease stages and may contribute to a more predictable PSMA expression profile prior to hormonal intervention. Tumor differentiation has also been reported as a key factor affecting PSMA expression. Transition to a neuroendocrine phenotype, frequently associated with combined TP53 and RB1 loss, is linked to markedly reduced or heterogeneous PSMA uptake [29]. Since these genetic alterations are less prevalent in primary Asian PCa, it is plausible that Asian patients initially present with more uniformly PSMA-avid disease compared to Western counterparts. As the disease progresses, however, the accumulation of diverse genomic alterations may lead to greater heterogeneity in PSMA expression over time.
  Furthermore, emerging evidence suggests that the tumor’s genetic profile can influence response to PSMA-targeted RLT. In Western cohorts, loss-of-function mutations in TP53 and RB1 have been associated with lower PSMA expression, resistance to radiation-induced damage, and poorer outcomes following ^177^Lu PSMA therapy [29]. These adverse genomic alterations appear to be less frequent in Asian patients, especially in early disease stages [10], implying that Asian patients might experience more favorable responses to PSMA-targeted RLT, at least initially. In addition to tumor suppressor genes, alterations in DNA damage repair (DDR) pathways have been explored as potential modifiers of PSMA expression. A translational study demonstrated that mCRPC tumors with deleterious BRCA2 or ATM mutations exhibited significantly higher membranous PSMA expression, suggesting that DDR-deficient tumors may be more amenable to PSMA-targeted approaches [36]. However, clinical findings remain inconclusive. In two retrospective cohorts, DDR alterations were not significantly associated with improved PSA response, PFS, or OS following PSMA-targeted RLT [37, 38]. Given the variable prevalence of DDR gene mutations across ethnic groups, including lower BRCA2 and ATM mutation rates reported in early-stage Asian PCa, further investigation is warranted to assess the clinical relevance of DDR status in guiding PSMA-targeted RLT across diverse populations [9, 10]. Recently, multi-omics analyses have demonstrated differences in drug metabolism among Asians, Blacks, and Whites with PCa, and such racial disparities in metabolic signatures may also contribute to varying sensitivity to systemic therapies [39]. The overall impact of other genomic factors such as FOXA1 and SPOP mutations remains unclear in the context of PSMA-targeted RLT.
3. Myelosuppression
  An important clinical factor to consider is the potential difference in bone marrow reserve and toxicity profiles in Asian patients. It is well documented that Asian patients are more prone to myelosuppression from cytotoxic chemotherapy than Caucasian patients [40, 41].​ For example, East Asian men treated with docetaxel experience significantly higher rates of grade 3–4 neutropenia and febrile neutropenia compared to Western trials. Real-world data from Hong Kong showed ~ 40–47% incidence of grade 3–4 neutropenia on docetaxel, versus ~ 12–32% in comparable Western studies [41]. A meta-analysis of 120 trials found that studies conducted in Asia had > 70% incidence of high-grade neutropenia, highlighting an increased sensitivity of Asian bone marrow to taxane toxicity, regardless of cancer type [42]. Although PSMA-targeted RLT is generally less myelosuppressive than multi-cycle chemotherapy, it still delivers radiation to the bone marrow, and cumulative hematologic toxicity remains a dose-limiting factor [43]. A key concern is that most patients receiving PSMA-targeted RLT are in the mCRPC setting, where they have typically undergone prior taxane chemotherapy and/or ARPI and multiple bone metastases. Many Asian patients may start PSMA-targeted RLT with already compromised bone marrow reserve. In addition, while classic chemotherapy doses take the patient body mass index into consideration, currently commercially approved PSMA-targeted RLT administer fixed doses, regardless of the patient’s body mass or tumor burden. Several factors have been identified as predictors of hematologic toxicity during PSMA-targeted RLT. These include baseline cytopenia, extensive bone marrow involvement by metastases, high total tumor volume on PSMA PET/CT, prior exposure to myelosuppressive therapies (such as taxanes), and impaired bone marrow reserve due to cumulative treatment burden [43–46]. Given these risk factors, careful patient selection is essential to minimize hematologic complications in Asian populations, who may have inherently lower marrow tolerance. Comprehensive assessment, including baseline blood counts, imaging evaluation of marrow involvement and review of prior treatment history, should guide eligibility and dosing strategies. While standard fixed-dose regimens are widely used, individualized approaches may be warranted in frail patients or those with borderline marrow reserve, and the patient’s receptiveness to transfusion should also be considered.
  In summary, while PSMA-targeted RLT appears safe in Asian populations, awareness of potentially lower bone marrow reserves, especially in heavily pre-treated mCRPC patients, is critical. Importantly, these concerns further support the rationale for exploring earlier use of PSMA-targeted RLT, before significant marrow suppression occurs, to maximize both safety and efficacy in Asian patients.

### Future directions

PSMA-targeted RLT is poised to become a standard treatment option in Asia, but several steps are needed to optimize its integration into clinical practice. Key points that will guide the future of PSMA-targeted RLT include the following.

1. Real-world Data
  Implementation of PSMA-targeted RLT have begun in East Asia. In Korea, PSMA-targeted RLT received regulatory approval in 2024, and patient treatments are now being conducted as part of standard clinical practice. Broader clinical adoption and potential reimbursement discussions are underway as the therapy gains momentum. China is actively employing PSMA-targeted RLT through domestic development and clinical trials. While national regulatory approval has not been granted yet, large-scale multi-center trials are ongoing, positioning China for potential approval in the coming years. A dedicated Japanese phase II bridging trial (NCT05114746) is underway to evaluate [^177^Lu]Lu-PSMA-617 in Japanese mCRPC patients​. This trial includes a safety run-in confirming tolerability of the standard 7.4 GBq dosing in Japanese patients, and features both post-taxane and pre-taxane cohorts to mirror real-world use. The results will likely facilitate regulatory approval in Japan and could guide dosing protocols tailored to Asian men.
  As more institutions across Asia adopt PSMA-targeted RLT into clinical practice, collecting real-world data will be most informative. Establishing an Asia-Pacific PSMA-targeted RLT registry or consortium for systematic collection of data on patient characteristics, efficacy outcomes, and safety profile will complement clinical trial data, uncover subtle ethnic differences, and provide insights into long-term or rare adverse events. Collaborative data sharing across countries will also allow for analysis of various subgroups with smaller patient volumes and enhance our understanding of PSMA-targeted RLT in Asian populations.
2. Novel Radiopharmaceuticals Developed in Asia
  Multiple new PSMA-targeted radioligands are emerging from Asia, promising expanded treatment options. In Korea, [^177^Lu]Ludotadipep, an albumin-binding PSMA agent, achieved ≥ 50% PSA declines in 37.5% of patients after a single low-dose administration [47], and a multi-cycle phase II trial also reported high disease control rate [EANM’24 Abstract, EJNMMI (2024), Volume 51,Suppl 1 page 5328]. Additionally, Cellbion developed [^177^Lu]Lu-DGUL, a novel PSMA-targeting agent, with phase II trial results expected later this year. In China, an Evans Blue–conjugated PSMA ligand, [^177^Lu]Lu-EB-PSMA-617, showed a ~ 57% PSA response rate with a median OS of ~ 12.6 months even at low 2.0 GBq dosing [48]. Notably, its efficacy per dose was comparable to outcomes with higher-dose [^177^Lu]Lu-PSMA-617 therapy. While these developments are encouraging, one must cautiously interpret cross-trial comparisons, as differences in molecular structure and pharmacokinetics (e.g. albumin-binding motifs prolonging tumor residence) can significantly impact tissue uptake and toxicity, and the methods of assessing dosimetry vary greatly.
3. Optimizing Therapeutic Efficacy
  As PSMA-targeted RLT becomes more widely available in Asia, optimizing patient selection is critical to maximize the therapeutic benefit while minimizing toxicity. Current evidence suggests that not all patients derive equal benefit from PSMA-targeted RLT, with factors such as PSMA uptake intensity, tumor burden, and baseline hematologic reserve influencing outcomes. Moreover, emerging genomic insights, such as poor responses in patients harboring TP53 or RB1 mutations, point toward the potential role of molecular profiling in guiding treatment decisions. Future research should focus on validating predictive biomarkers to better identify Asian patients who are likely to achieve durable responses.
  In addition, combination strategies offer promising avenues to enhance efficacy [49]. Recent evidence also supports the potential benefit of combining PSMA-targeted RLT with ARPI. In the randomized ENZA-p trial, the addition of [^177^Lu]Lu-PSMA-617 to enzalutamide significantly prolonged PSA PFS compared to enzalutamide alone (13.0 vs. 7.8 months, HR 0.43, *p* < 0.0001), without excess severe toxicity [50]. Observational and case-based studies from Asia further suggest enhanced response rates and improved survival when PSMA-targeted RLT is combined with abiraterone acetate or enzalutamide, even in genomically high-risk patients, such as those with ATM mutations [51–53]. Trials investigating the synergy between PSMA-targeted RLT and PARP inhibitors, particularly in patients with DNA damage repair deficiencies, are ongoing and could be extended to include Asians or replicated in Asian cohorts [54]. Other approaches, such as sequencing PSMA-targeted RLT with chemotherapy, or incorporating supportive measures like bone-protective agents and growth factors in high-risk patients, warrant further exploration as methods of improving long-term outcomes.
  Alpha-particle emitters such as Actinium-225 (Ac-225) and Astatine-211 (At-211) are gaining attention due to their higher linear energy transfer and shorter tissue penetration range, which may offer improved efficacy against micrometastatic disease and reduced off-target toxicity compared to beta-emitters like Lu-177 [55]. Recent Asian studies have demonstrated promising outcomes using [^225^Ac]Ac-PSMA-617 in heavily pretreated mCRPC patients, with PSA response rates exceeding 60–70% and acceptable safety profiles despite prior Lu-177 therapy exposure [56, 57]. Notably, xerostomia and hematologic toxicity remain common side effects and require mitigation strategies. Additionally, first-in-human study of [^211^At]PSMA-5 has been reported from Japan, indicating the feasibility of At-211–based targeted alpha therapy in clinical settings [58]. Although further prospective trials are warranted, these alpha-emitting approaches represent a promising therapeutic option in patients with aggressive or treatment-refractory disease.
4. Expanding PSMA-targeted RLT To Earlier Disease Settings
  There is growing interest in expanding the use of PSMA-targeted RLT beyond heavily pre-treated mCRPC to earlier stages of PCa. In chemotherapy-naïve mCRPC [24–26, 59], as first-line in mCRPC [50, 60], and in mHSPC settings [61–63]. In particular, early clinical studies in mHSPC have demonstrated encouraging efficacy and safety of PSMA-targeted RLT. A single-arm pilot study in patients with synchronous high-volume mHSPC ineligible for chemohormonal therapy reported PSA50 response in 90% and undetectable PSA in 50% of patients, with no grade 3/4 adverse events [61]. The randomized phase 2 UpFrontPSMA trial demonstrated that [^177^Lu]Lu-PSMA-617 followed by docetaxel significantly increased the rate of undetectable PSA at 48 weeks (41% vs. 16%, *p* = 0.002) compared to docetaxel alone, without additional toxicity [62]. Furthermore, the CONSOLIDATE trial evaluated [^177^Lu]Lu-PSMA-617 as consolidation after docetaxel in patients with residual disease and showed higher PSA suppression (60% vs. 13%), improved radiographic response (53% vs. 7%), and prolonged PFS without grade 3/4 toxicity [63]. These findings suggest that early integration of PSMA-targeted RLT may enhance treatment response and disease control in mHSPC, particularly in high-volume disease. Ongoing trials such as PSMAddition in mHSPC and PSMA-DC in oligometastatic disease are further evaluating the role of early PSMA-targeted RLT across diverse clinical settings. Given that Asian patients frequently present with aggressive, high-burden disease at diagnosis, early integration of PSMA-targeted RLT may be particularly advantageous in this population by addressing the high metastatic potential before resistance mechanisms emerge.
  To ensure that the changing strategies in PCa care are globally applicable, it is vital that future trials enroll sufficient numbers of participants of Asian ethnicity, as well as other underrepresented groups to generate representative data regarding the efficacy and safety of PSMA-targeted RLT. Furthermore, investigational use of PSMA-targeted RLT in neoadjuvant settings or as an adjunct to local therapy for high-risk localized PCa presents exciting research opportunities. Active participation of Asian centers in such trials will be key to defining the role of PSMA-targeted RLT across the full spectrum of PCa.

## Conclusions

PSMA-targeted RLT holds great promise for PCa patients across Asia. While the limited clinical outcomes in Asian patients appear comparable to those observed in Western populations, factors such as more aggressive disease at presentation, varying genomic alteration profiles, and increased susceptibility to hematologic toxicity underscore the need for strategies adapted for Asian men.

Moving forward, accumulating robust real-world data, advancing region-specific clinical trials, and integrating novel radiopharmaceuticals will be critical to optimizing PSMA-targeted RLT for Asian patients. Emphasis should be placed on refining patient selection criteria, exploring effective combination therapies, and expanding the use of PSMA-targeted RLT into earlier disease settings—particularly given the high-risk features frequently observed at diagnosis in this population. Ensuring adequate representation of Asian cohorts in clinical trials will lead to evidence-based guidelines of global relevance.

Ultimately, a personalized approach grounded in greater understanding of the ethnic-specific disease biology and treatment response, will be key to maximizing the therapeutic potential of PSMA-targeted RLT across Asia. Continued collaboration among clinicians, researchers, and healthcare systems will drive the progress toward more effective and safer PCa care tailored to the needs of Asian patients.

## Data Availability

Data sharing not applicable to this article as no datasets were generated or analyzed during the current study.
